# Disturbance and the (surprising?) role of ecosystem engineering in explaining spatial patterns of non‐native plant establishment

**DOI:** 10.1002/ece3.7915

**Published:** 2021-08-13

**Authors:** Meredith Root‐Bernstein, César Muñoz, Juan J. Armesto

**Affiliations:** ^1^ CNRS Musée National d’Histoire Naturelle Paris France; ^2^ Instituto de Ecología y Biodiversidad Santiago Chile; ^3^ Center for Sustainability and Applied Ecology Santiago Chile; ^4^ Department of Ecology Pontificia Universidad Católica de Chile Santiago Chile

**Keywords:** bioturbation, disturbance, ecosystem engineering, herbivory, non‐native plants, *Octodon degus*

## Abstract

Different conceptions of disturbance differ in the degree to which they appeal to mechanisms that are general and equivalent, or species‐, functional group‐, or interaction‐specific. Some concepts of disturbance, for example, predict that soil disturbances and herbivory have identical impacts on species richness via identical mechanisms (reduction in biomass and in competition). An alternative hypothesis is that the specific traits of disturbance agents (small mammals) and plants differentially affect the richness or abundance of different plant groups. We tested these hypotheses on a degu (*Octodon degus*) colony in central Chile. We ask whether native and non‐native forbs respond differently to degu bioturbation on runways versus herbivory on grazing lawns. We ask whether this can explain the increase in non‐native plants on degu colonies. We found that biopedturbation did not explain the locations of non‐native plants. We did not find direct evidence of grazing increasing non‐native herbs either, but a grazing effect appears to be mediated by grass, which is the dominant cover. Further, we provide supplementary evidence to support our interpretation that a key mechanism of non‐native spread is the formation of dry soil conditions on grazing lawns. Thus, ecosystem engineering (alteration of soil qualities) may be an outcome of disturbances, in which each interacts with specific plant traits, to create the observed pattern of non‐native spread in the colony. Based on these results, we propose to extend Jentsch and White (Ecology, 100, 2019, e02734) concept of combined pulse/ disturbance events to the long‐term process duality of ecosystem engineering/ disturbance.

## Graphical abstract

Non‐native plant species accumulate on the centers of native degu (*Octodon degus*) colonies. We show that this is the result of the interaction and accumulation of two kinds of disturbances (biopedturbation and herbivory) with different effects on plant species over time. Although disturbance is usually represented as different from, or indeed opposite to, ecosystem engineering, we show that ecosystem engineering best explains the outcome of these two disturbances over time.

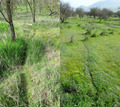

## 1 INTRODUCTION

Disturbances and perturbations are key factors determining change in vegetation composition over time (Pickett & Cadenasso, 2005). Although community ecology research has largely shifted from explaining community structuring to explaining the consequences of biodiversity for ecosystem functioning, this is not because community structuring has been fully explained (Aviolo et al., 2019; Jentsch & White, 2019). An open question for both ecosystem function questions and community structuring questions is the degree to which the mechanisms underlying them are general and equivalent, or species‐, functional group‐, or interaction‐specific (Aviolo et al., 2019). For example, a distinction is sometimes made between perturbation, used to describe exogenous or trophic effects, and disturbance, meaning endogenous or nontrophic effects (Proulx & Mazumender, 1998). Other ecologists consider both trophic and nontrophic disturbances as equivalent forms of disturbance (Mackie & Curry, 2001). For example, the mechanisms of the intermediate disturbance hypothesis (IDH) formally imply that all forms of disturbance (e.g., trophic and nontrophic) equivalently allow some species to escape competition due to the destruction of other species’ biomass: This assumption of equivalence might be one among other reasons why the IDH is not predictive (Shea et al., 2004; Willig & Presley, 2018). However, a possible interpretation of Connell's (1978) version of the IDH is that only *selective* disturbances that target *dominant* species lead to peak levels of diversity (although Connell 1978 made predictions about proportionate abundance, not diversity). However, there are multiple definitions of plant species dominance, which confuse this interpretation (Aviolo et al., 2019). A more complex and useful new conceptualization of disturbance has been offered by Jentsch and White (2019). They proposed that all disturbances (trophic and nontrophic) are simultaneously pulses, as every pulse/ disturbance is a multifactorial event that increases some variables, decreases other variables, and perhaps does not affect another set of variables. This implies that different pulse/disturbance events produce *unique combinations* of multifactorial effects.

In line with Jentsch and White’s (2019) multifactorial pulse/ disturbance event concept, some data demonstrate that the identities of the disturbing and disturbed species can affect disturbance outcomes, which should be expected if each kind of pulse/ disturbance produces a different set of increases and decreases in a unique set of variables. The literature on non‐native herb establishment or invasion provides some examples of nonidentical effects of trophic or nontrophic disturbance. For example, species identity—native or non‐native—of both the disturbing (animal) and establishing (plant) species has been shown to lead to different trajectories of plant community composition (e.g., invasion of non‐native plants) (Parker et al., 2006). Soil disturbance by a native small mammal, but not herbivory by invasive herbivores, leads to expansion of a non‐native herbaceous plant in central Chile (Torres‐Díaz et al., 2012). A plausible explanation for how the identities of interacting species drive plant community change is that trophic and nontrophic disturbance effects are highly sensitive not only to the type of disturbance (e.g., selective herbivory versus unselective herbivory versus mounds versus runways) but also to the timing, spatial distribution, scale, etc., in which a given disturbance is carried out by different species (Maschinski & Whitham, 1989). Plant species tolerate and respond to these disturbances differently, with different competitive advantages (Grace, 1991). For example, non‐native plants are often ruderals adapted to intensive grazing, making them potentially superior competitors to native plants under herbivory and large mammal trampling (Fraser & Madson, 2008; Keane & Crawley, 2002; Schiffman, 1994; Seabloom et al., 2009).

Nontrophic disturbances of small mammals such as burrow digging have attracted attention since the origins of ecology (Kelt, 2011; Whitford & Kay, 1999). In addition, long‐term studies have examined trophic impacts of small mammals on plant community change, for example, in kangaroo rats (Brown et al., 2001). Small mammal trophic and nontrophic disturbances frequently lead to increases in plant richness and diversity (Root‐Bernstein & Ebensperger, 2012). Native small mammal disturbances can also present a threat to native herbaceous plant communities by favoring grazing‐adapted non‐native plants (Torres‐Díaz et al., 2012).

**FIGURE 1:**
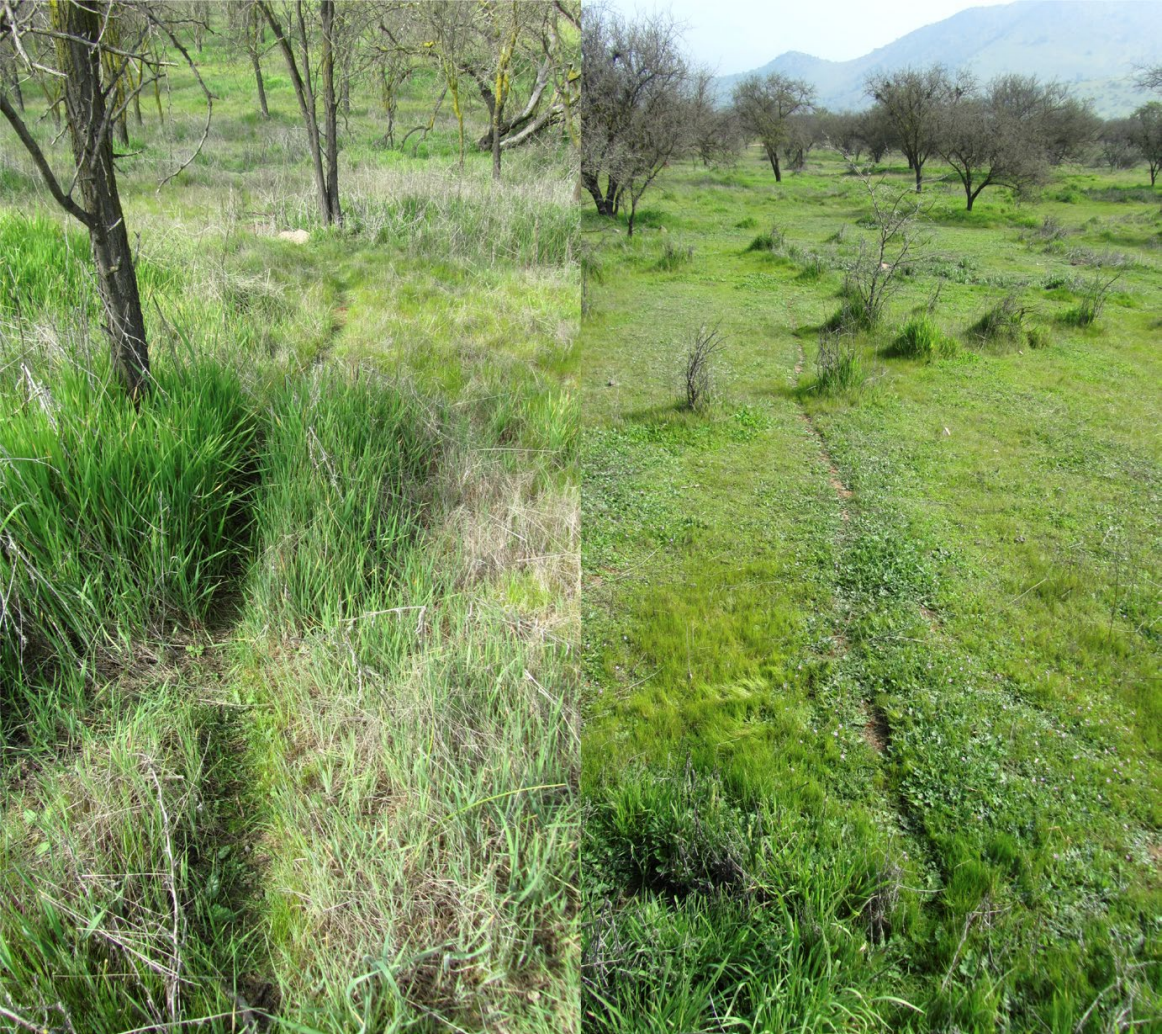
Left, a very new degu runway formed by pushing the grass down or aside. The tall grass shows no signs of grazing, which probably reflects the very recent establishment of the burrow at the end of the runway. Right, more established degu runway in a degu grazing lawn. Both photographs are taken during 2013 in early spring. Photos © MR‐B

In this study, we ask whether native and non‐native herbaceous plants respond differently to degu runway‐related biopedturbation (running up and down runways) and to degu herbivory. The degu *Octodon degus* is a group‐living, colonial burrow‐dwelling rodent in central Chile (Ebensperger et al., 2019) that creates runways between burrow entrances and grazes aboveground (Figure 1; Madrigal et al., 2011). Their grazing lawns on colonies increase herbaceous plant richness along with other elements of biodiversity (Root‐Bernstein et al. , 2013, 2014). Degu colonies exhibit central areas with high runway density and multiple‐entrance burrows <10 m apart, as well as extensive, less‐dense peripheries constructed by dispersing juveniles and occupied during high degu‐population years (Ebensperger et al., 2009; Ebensperger, Chesh, Castro, Tolhuysen, Quirici, Burger, Sobrero, et al., 2011; Ebensperger, Chesh, Castro, Tolhuysen, Quirici, Burger, & Hayes, 2011; Quirici et al., 2011). Biopedturbation is concentrated on runways especially before grazing lawn creation, but grazing occurs both on and off runways (Root‐Bernstein et al. in submission). Surrounding grassland is expected to be mainly annual herbs and at least half non‐native (Deil et al., 2007).

We predict that different species will respond positively (increase in abundance/ cover) to different combinations of disturbances forming different disturbance/ pulse conditions, represented in the degu colony as areas with differing accumulation of herbivory or biopedturbation, or both, over time. We expect that herbivory may vary in intensity/ accumulated impacts both across the colony structure and at the level of structures within the colony (on and away from runways). This pattern is not necessarily the same as the distribution of the highest biopedturbation, which we expect to be diffused across colony center runways and grazing lawns, and intensively concentrated along runways in more peripheral areas. These patterns may interact, although *a priori* we are unsure whether biopedturbation level is always correlated with herbivory level or only in specific spaces. We expect accumulated disturbance/ pulse outcomes from both herbivory and biopedturbation to be jointly highest in the older colony center. Specifically, we predict that the ruderal non‐native herbs should be more common where bioturbation on runways is the highest. We also predict that non‐native herbs adapted to herbivory should be more common at sites with higher grazing pressure (Holmgren et al., 2000). Finally, how a dominant species reacts to a disturbance/ pulse regime will impact how competitor species experience and react to the disturbance/ pulse regime. We thus predict that the impacts of trophic and nontrophic disturbance on the dominant (in terms of cover) native taxon, grasses, may mediate increases in non‐native herbs (del Pozo et al., 2006; Holmgren et al., 2000).

## 2 MATERIALS AND METHODS

### 2.1 Study site

The study took place in September and October 2011 at the Estación Experimental Rinconada de Maipú (33°23′S, 70°31′W, altitude 495 m), a field station of Universidad de Chile, Santiago, Chile. The field station consists of espinal (*Acacia caven* savanna) subject to occasional fires (1–2 events per decade) and grazing by cattle and sheep (<1 sheep per hectare over almost 900 ha; C. Araneda Pers. Comm. 2014), open grasslands dominated primarily by native perennial grasses, and denser matorral (evergreen shrubland), dominated by sclerophyllous shrubs and perennial herbs. The study site includes extensive degu colonies with lawns of herbaceous species, found essentially in the valleys of a small mountain range (<1,000 m in elevation) forming one side of the field station.

### 2.2 Plot selection

**FIGURE 2:**
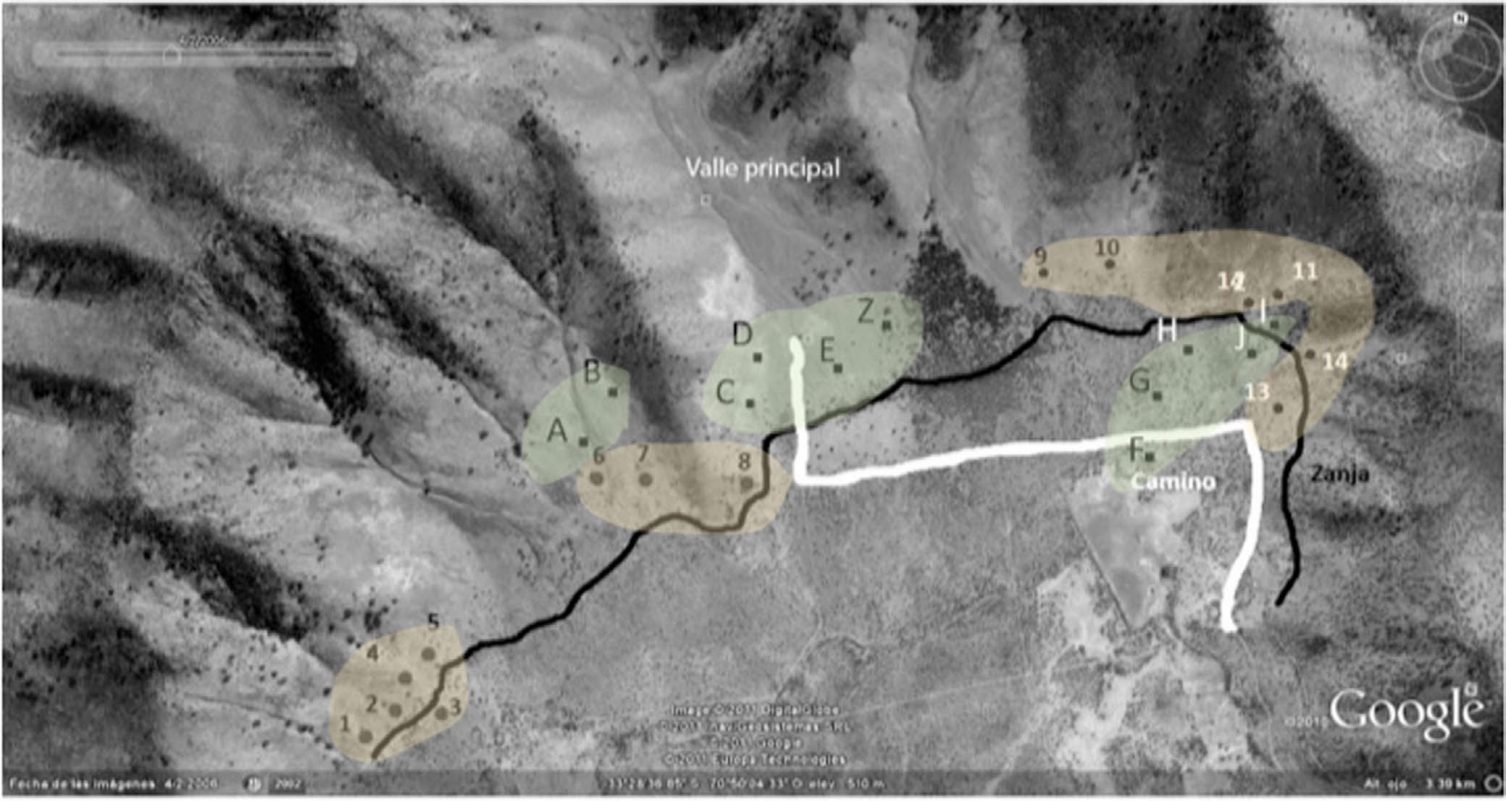
Field site with the central and peripheral plots marked. The letters highlighted in green are the ten central plots, and the numbers highlighted in yellow are the thirteen peripheral plots. The white line represents an access road, and the black line represents a dry ditch

We set up 13 “peripheral plots” and 10 “central plots” on the degu colony (Figure 2). Central plots were defined as 10 m × 10 m squares containing >20 degu runways. Central plots were separated by at least one burrow system. Peripheral plots were defined as 10 m × 10 m squares with <5 degu runways. Note that while the runway shown on the left of Figure 1 is peripheral, the peripheral plots in the study did not consist of brand‐new runways or burrows that had just been established, like the one shown in the figure. There was thus some previous history of biopedturbation and herbivory of unknown temporal depth. The colony center–periphery distance at our field site is a radius of a minimum of approximately 300 m. Additional selection criteria for plots included evidence of fresh soil from recent excavation at burrows in or adjacent to the plot, and little or no evidence of rabbit droppings or coruro (*Spalacopus cyanus*) mounds. We considered the presence of fresh soil from excavations or the presence of fresh droppings to determine that a burrow was occupied (Ebensperger, Chesh, Castro, Tolhuysen, Quirici, Burger, & Hayes, 2011; Ebensperger, Chesh, Castro, Tolhuysen, Quirici, Burger, Sobrero, et al., 2011). There were no rabbit burrows within the degu colony. None of the plots included coruro mounds. Plots were oriented so that they were bisected by one or more degu runways forming a transect across the plot.

### 2.3 Disturbance accumulation proxies

Our methods are based on a form of space‐for‐time substitution (Pickett, 1989). Following the advice of Pickett for appropriate design of space‐for‐time substitutions, we selected several space‐for‐time proxies, drawing on our knowledge of the local vegetation community change process on degu colonies (Root‐Bernstein et al., 2014). We also draw on long‐term studies of prairie dog (*Cynomys ludovicianus*) colonies, since this species has a similar life history, ecological niche, and use of habitat, compared to degus. Prairie dog colonies expand outwards so that central areas are older than peripheral areas (Whicker & Detling, 1989). Since we have observed similar patterns for degus (see Introduction), our first proxy for disturbance accumulation is the location, peripheral or central, of the plots examined. Over time, cover of tall grass in prairie dog colonies declines and is replaced by low herb cover, starting in the colony center and moving outward to the periphery (Archer et al., 1987; Garrett et al., 1982). In central Chile, grasses are also dominant taxa that decline under herbivory (del Pozo et al., 2006; Holmgren et al., 2000). We therefore expect grass cover to be negatively correlated with age of colony sections, as well as potentially mediating the increase in other herb species through reduction in competition. A third proxy is derived from field observations at our research site: Moss is common on runways and may be an early colonizer of runways. We thus used moss cover on runways as a third proxy for accumulated disturbance over time.

### 2.4 Herbaceous plant data

Along each runway‐transect, we collected quadrat data at two distances from the runway‐transect: over the runway (“on runway”) (runways are about 8 cm wide) and 25 cm from the runway edge (“off runway”). Each quadrat was a square cardboard frame of 10 cm^2^, which we laid on the ground over the sample and photographed with digital cameras in autofocus mode at a distance of approximately 1 m from the ground. Five quadrats of on‐runway samples were recorded every 2 m along the runway‐transect at odd‐numbered meter marks. Five quadrats of off‐runway samples were recorded at even‐numbered meter marks. Quadrats alternated to the left and right of the transect.

### 2.5 Plant identification

Plant species rarely completely overlapped each other due to an absolute low abundance in all plots, and it was possible to identify each species from the photographs of the quadrats by the shape of cotyledons, leaves, and flowers. Names and distributions are according to Hoffmann (1978), except for mosses and grasses. We classified mosses as endemic following Larraín (2009), which indicated that the majority of mosses found in Chile are native to Chile. We were not able to identify grasses to species, but grasses were assumed to be mainly native or endemic species (Finot et al., 2011). Whenever herbs could not be identified by species, they were classed according to morphospecies according to cotyledon, colors, and leaf shapes.

Plants were identified and counted using SamplePoint (in R), which records the classification into user‐defined categories for pixels at crosshairs arranged in a regular grid over the digital photograph. We set our grid to 25 crosshairs per photograph. Dead plants were not counted. When plants overlapped, we counted the top (visible) species only. Overlap occurred only rarely (estimated <10% of all crosshairs). Cover was calculated as count number.

### 2.6 Measures of herbivory

We assessed herbivory by measuring the amount of rolled oats eaten from dishes. Oats are a favorite food of degus and are used to trap them (e.g., Ebensperger, Chesh, Castro, Tolhuysen, Quirici, Burger, & Hayes, 2011; Ebensperger, Chesh, Castro, Tolhuysen, Quirici, Burger, Sobrero, et al., 2011; Quirici et al., 2011). During the study season (spring), degus graze diurnally, alone or in loosely associated foraging groups (Lagos et al., 2009). We placed small metal dishes 11 cm in diameter in each plot, near the degu runway‐transect. Dishes were filled with 25 g of rolled oats in the mornings of 3/10/11, 16/10/11, and 23/10/11. Dishes were checked and weighed with an electronic weight (Acculab GS‐200) 24 hr later. The amount of oats eaten was calculated as the difference between the weight of oats with which the dish was filled the previous day, and the current weight. In some cases, the dishes of oats gained up to 2 g of water from dew. The amount eaten is thus precise to within ≤2 g. We did not observe ant activity at the oat dishes. Spillages were noted and not treated as eaten. Just prior to data collection, another experiment started in the same research site, which involved baiting Sherman traps with oats, and some of these traps were close to one of our central plots. Thus, we did not collect foraging data from this plot.

### 2.7 Measuring bioturbation on runways

We measured bioturbation on runways as the amount of degu traffic along the runway‐transect in each plot. We recorded degu traffic using tracking cards (Meserve, 1981). We cut strips of rag paper approximately 32 cm × 7 cm (to fit within a degu runway). A central square on the paper strip about 7 cm^2^ was colored in with a black hard pastel. Two such tracking cards were placed end to end along the runway‐transect in each plot and fixed in place with nails. Tracking cards were put in place on 17/10/11 and collected on 27/10/11. Collected tracking cards were sprayed with a fixative for pastels to prevent smudging. Tracking cards were photographed, and the images were analyzed in Adobe Photoshop^®^, using the “count” function. We counted discrete toe and palm marks (“footmarks”). We summed the total number of footmarks for the two tracking cards for each site to yield a measure of bioturbation along runways. We observed only one footprint from another species, not identified. We did not collect bioturbation data for the site omitted from the foraging data collection.

### 2.8 Statistical analysis

Native and non‐native plant abundances or cover was approximated by total count number from the virtual grids in SamplePoint, summed across the quadrats of each plot; we summed across different plant categories and/or quadrat positions to construct plot‐level measures. Grass cover was calculated separately in the same way. Biopedturbation was quantified as the sum of footmarks per plot. Herbivory was quantified as mean grams of oats eaten per plot across the three sampling periods. We compared these across central and peripheral plots and along the gradient of grass cover. Because space‐for‐time substitutions involve interpretive assumptions that are not present in long‐term manipulative experiments, we chose to use the most conservative (least powerful) statistical approaches available to decrease the likelihood of detecting ecologically weak effects (Amrhein et al., 2019). Our comparisons thus used nonparametric tests (Kruskal–Wallis and chi‐square) to compare native and non‐native forb cover, and the distribution of these plant categories across on and away from runways and in the center or periphery plots. We used the Student *t* test and correlations to examine grass distribution, the relationship between the measures of herbivory and biopedturbation with grass cover and moss cover, and the distribution of native and non‐native plants.

## 3 RESULTS

### 3.1 Plant community description

We observed 10 native or endemic forb species or morphospecies, two taxonomic groups dominated by native species (mosses and grasses) and 3 non‐native species along all of our transects. There was no significant difference in cover per taxa between native and non‐native forbs (Kruskal–Wallis test, H = 0.092, *df* = 1, *p* = .756).

### 3.2 Native and non‐native herb distributions

**FIGURE 3:**
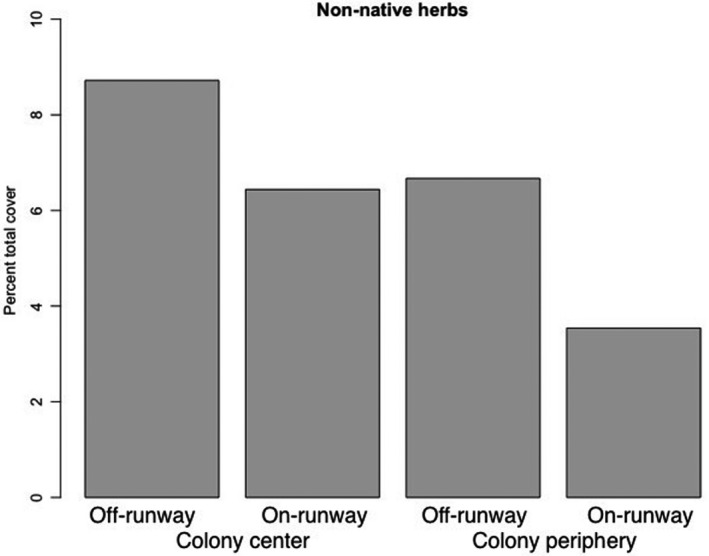
Distribution of non‐native herbs across colony areas and on and off runways, expressed as a percent of the total cover available in each category

Natives and non‐natives were distributed differently across the plot locations, with non‐native cover higher in central plots and native cover higher in peripheral plots, which was significantly different from the expected cover distribution (χ^2^ test, χ^2^ = 177.73, *df* = 3, *p* = 2.2 × 10^–16^). At a finer scale, natives and non‐natives were also distributed differently on or away from runways: Native cover was higher off runways than on, and higher than non‐natives; non‐natives were also higher off runways (χ^2^ test, χ^2^ = 232.84, *df* = 3, *p* = 2.2 × 10^–16^). We found an interaction between colony location (center/ periphery) and runway location (on/ off) on the total cover of non‐native species: The cover of non‐natives is higher in the colony center than in peripheries, and higher on runways than off runways, a distribution significantly different from expected (Figure 3; χ^2^ test, χ^2^ = 61.5, *df* = 3, *p* = 2.8 × 10^–13^).

### 3.3 Space‐for‐time proxies

Mean grass cover on off‐runway quadrats, the proxy for colony age, was not lower in central plots, differing from our expectation (Student's *t* test, t = 0.193, *df* = 15.98, *p* = .84). The ratio of non‐native to native herb abundance per plot was negatively correlated with the grass proxy (mean grass cover off runway) per plot (*r* = −.684, *df* = 19, *p* = .0006). Focusing on moss as a proxy of colony age, we find that the mean percent cover of moss differed significantly across on–off and center–periphery and was highest on peripheral runways (χ^2^ test, χ^2^ = 38.03, *df* = 3, *p* = 2.785 × 10^–08^).

### 3.4 Grass distribution as a mediator of competition with other herbs

The ratio of grass cover on to off runways per plot was not correlated to the general distribution of other herbs on and off runways per plot (*r* = .073, *df* = 19, *p*‐value = .75). Although grass tended to have relatively greater cover on runways, and other herbs tended to have relatively greater cover off runways, these differences were not correlated at the plot level.

### 3.5 Herbivory and bioturbation on runways

The average amount of oats eaten per day did not differ between peripheral and central plots (Student's *t* test, t = 1.31, *df* = 21, *p* = .203). The number of footmarks registered on runways also did not differ between peripheral and central plots (Student's *t* test, t = 0.54, *df* = 21, *p* = .592). Footmarks and total amount of oats (on and off runways) eaten in each plot were highly correlated (*r* = .99, *df* = 19, *p* < .0001). Footmarks increased as the percent cover of grass on *off‐runway* quadrats increased (*r* = .508, *df* = 21, *p* = .0134). However, there was only a weak, nonsignificant relation between percent grass cover *on runways* and footmarks (*r* = .262, *df* = 19, *p* = .251). Biopedturbation was not correlated with the ratio of non‐native to native herbs (*r* = −.189, *df* = 18, *p* = .43).

## 4 DISCUSSION

We found that the richness of native nonwoody herbaceous taxa (*N* = 11) was almost four times that of non‐native species (*N* = 3) on degu colonies. The number of native or endemic species may have been underestimated as we were not able to identify mosses or grasses to species level. As expected, the cover of non‐native plants was higher in the center of the colony than in the periphery. At a finer scale, the runways also influenced the distribution of native and non‐native plants. Non‐native plants had higher cover on runways in central plots than in peripheral plots.

We find at least partial support for our interpretation of peripheral colony sites as having been formed more recently and/or occupied more sporadically over time compared with colony centers. The grass proxy for accumulated disturbance did not lend support to the center–periphery split, with patches of grass found in both peripheral and central plots. Moss, as expected, had different distributions across central and peripheral plots. Although we expected moss to increase in extent over time on runways, we interpret the result to mean that moss is an early colonizer of runways created through biopedturbation (as expected), but reduces in extent as the microhabitat becomes drier (see below).

The distribution of high and low biopedturbation and herbivory was not explained by colony position, but was explained by grass cover off runways. As grass cover in the off‐runway quadrats increased, foot traffic on runways and amount of herbivory on oats increased. Grass avoidance (i.e., avoiding walking through grass and rather walking around it along runways) may thus mediate degu biopedturbation on runways. We suggest that avoiding grass will occur whenever it is long enough that it needs to be physically pushed aside to make a runway or impedes degu movement (e.g., Figure 1); on grazing lawns, the height of the plants does not impede movement and degus frequently run or walk across the lawns (Root‐Bernstein et al. in submission). We did not measure grass height in this study, and we have no data on the vegetation height at which degus no longer prefer to walk over/ through it.

Grass was also expected to mediate plant composition change through herbivory on grass reducing its competition with other herbs. Indeed, as grass cover decreased off runways, the relative cover of non‐native species increased. Although degus eat both grasses and forbs, including the non‐native forbs we observed in this study (Quirici et al., 2010), the persistence of grass along runway edges suggests that it is not opportunistically eaten (unlike the oats). In addition to eating grasses, degus also gather them to line their nests in spring, which requires long grass of the kind found around woody plants or away from grazing activity (pers. obs. MR‐B; Figure 1). Thus, while grass is undoubtedly a resource for degus, and its reduction leads to increases in relative cover by non‐native species, it is not entirely clear that degus selectively or substantially reduce grass cover through herbivory. It is possible that grass is reduced to a large extent or even primarily through indirect mechanisms, as we discuss below.

Our interpretation of the temporal process resulting in the observed spatial pattern of non‐native herb distribution is that grass reduction off runways over time leads to increased non‐native populations and the eventual colonization of the runway edge by non‐natives. However, it is not clear that eventual grass reduction on runways is what allows non‐natives to colonize runway edges in the center of the colony, since the grass did not reliably disappear in the colony center and was uncorrelated with other herb distributions relative to runway structure. Thus, non‐native expansion to runway edges may not be mediated by competition with grass, but by some other mechanism.

Ecosystem engineering effects on the soil may help explain how the increase in non‐natives first seen off runways spreads to runway edges. Runways are exposed to the sun. Grazing lawns, with vegetation only ~2–5 cm high, can be expected to lead to lowered evapotranspiration and less water being drawn into the soil by plant roots. These effects may lead to drier soils: The loss of or lack of moss in central runways may also point to drier soils in colony centers. Although we did not measure soil moisture in this study, as we did not anticipate its importance, other researchers have measured soil characteristics at the same research site. Bauer et al. (2013) show that soil penetrability, directly related to soil moisture (Ebensperger & Bozinovic, 2000), is highest at sites with shade cover and lowest at sites with the most bare earth (see also Ovalle & Avedaño, 1984). This bare earth would mainly correspond to areas of degu biopedturbation. Ebensperger and Bozinovic (2000) also show that in colony centers, soil moisture declines dramatically during the low‐precipitation months (summer and autumn). At the same time, where the grass is less productive due to being kept short through herbivory, this may lead directly to a reduced competitive advantage of grass. In fact, the two most abundant of the three non‐native species observed at our field site, *Camissonia* sp. and *Erodium cicutarium*, are considered to be desert‐adapted species and poor competitors with grass (Ehleringer et al., 1979; Holmgren et al., 2000; Schutzenhofer & Valone, 2006; Stamp, 1984), while only *E. moschatum*, which was much less abundant, is considered ruderal (IUCN Invasive Species Database, http://www.iucngisd.org/gisd/species.php?sc=518). If an important impact of degus is via soil aridity in grazing areas, this might also account for Madrigal et al. (2011) finding that the degu presence at their site resulted in a numerical dominance of non‐native species. Their research site is more arid than ours, and a further increase in soil aridity may more strongly favor non‐native desert‐adapted herbs.

**FIGURE 4:**
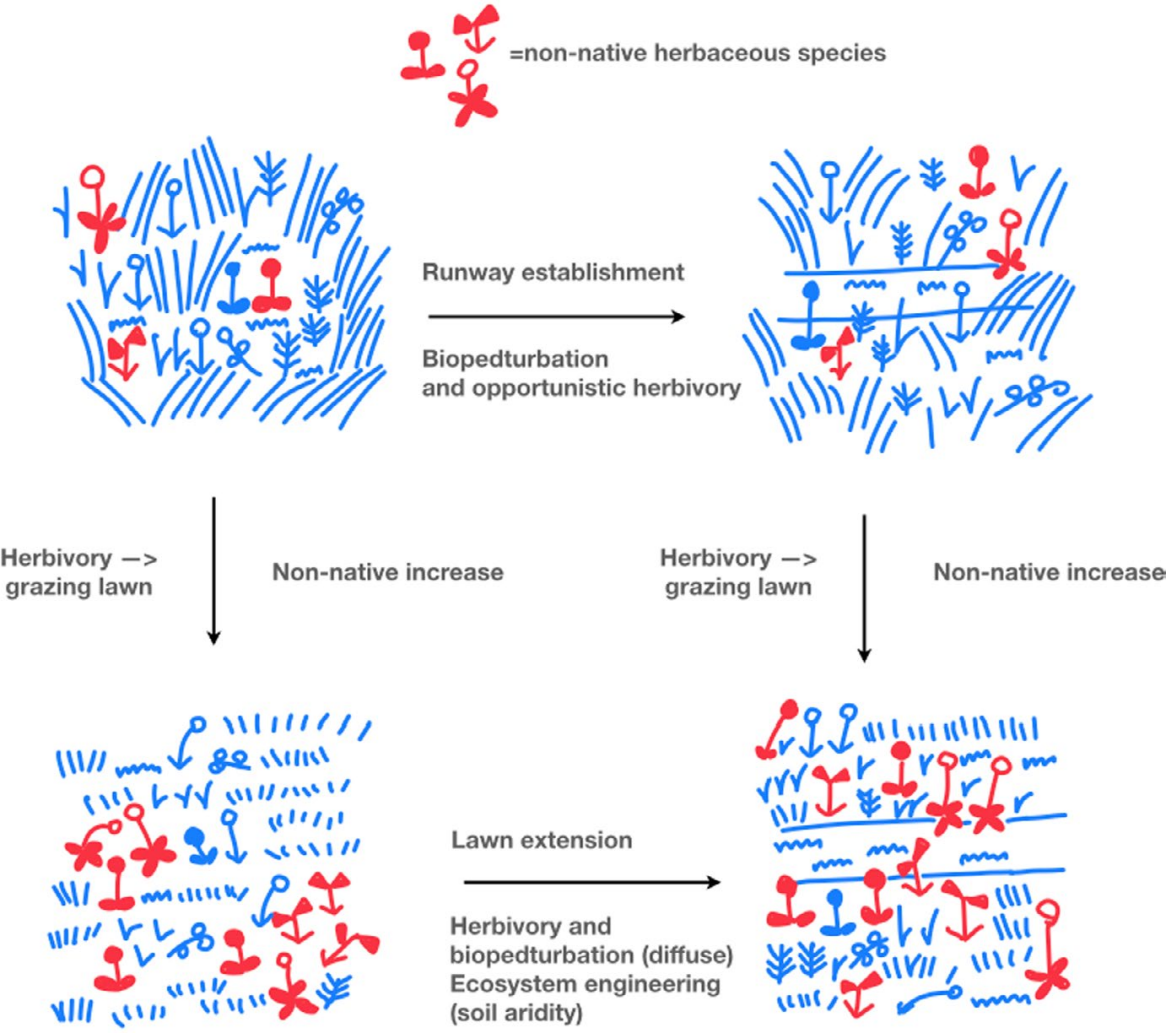
Diagram of the mechanisms of disturbance and their accumulation over time into an ecosystem engineering effect, which favors non‐native dry‐adapted species

In summary, nontrophic (biopedturbation) and trophic (herbivory) forms of disturbance/ pulse appear to have different impacts that interact with one another and with specific plant traits to create ecosystem engineering that favors non‐native herbs on degu colonies (see Figure 4). We found the least evidence for impacts of disturbance via biopedturbation. Biopedturbation clearly creates the runways, which appears to initially favor mosses. However, ongoing current biopedturbation disturbance effects could not clearly be separated from grazing effects as they were highly correlated. Further, only the least‐abundant non‐native herb found is ruderal. Trophic disturbance in the form of grazing effects might be interpreted from the reduction in grass, relative to other herbs, off runways, associated with increasing non‐native plants. We did not anticipate that ecosystem engineering affecting soil properties (soil aridity) might explain the expansion of non‐native species, since the majority of literature on non‐native herbs in central Chile focuses on their adaptations to herbivory rather than aridity, and disturbance literatures focus on the mechanisms of competition and biomass loss, without linking to ecosystem engineering. However, preexisting measurements of soil hardness and moisture at the same degu colony, and the desert adaptations of the most abundant non‐native species, provide evidence for the hypothesis that ecosystem engineering of the soil conditions contributes to the spread of non‐native herbaceous species on degu colonies.

In general terms, these results further expand the notion of pulse/ disturbance developed by Jentsch and White (2019). Not only are pulse/ disturbance events multifactorial, but also they have multitemporal impacts that develop over time in ways that do not simply reduce competition or increase biomass loss of dominant species (they may not do this at all), but also may accumulate or interact to form what we call ecosystem engineering. Wilby et al. (2001) come to a similar conclusion in a study of desert porcupines. However, the vast majority of other studies either do not mention both concepts together or contrast the supposedly independent and indeed *opposite* processes of disturbance and ecosystem engineering (e.g., Soissons et al., 2019). This conception of ecosystem engineering as opposite to disturbance can partly be understood from the perspective where pulses are also opposite to, rather than dimensions of, disturbance. The lack of integration of ecosystem engineering ecology with disturbance ecology also simply reflects the different and nonintegrated literatures from plant ecology, animal ecology, and plant–animal interaction ecology (see Root‐Bernstein, 2013). Further developing the conceptual link between pulse/ disturbances and ecosystem engineering offers an interesting direction for the development of better‐integrated theories of community structure.

## CONFLICT OF INTEREST

The authors report no conflicts of interest.

## AUTHOR CONTRIBUTIONS

**Meredith Root‐Bernstein:** Conceptualization (lead); Investigation (lead); Methodology (lead); Writing‐original draft (lead); Writing‐review & editing (lead). **Cesar Muñoz:** Investigation (supporting). **Juan J. Armesto:** Supervision (lead).

## Data Availability

Data are available on Dryad at https://doi.org/10.5061/dryad.vq83bk3sv.
